# The History and Capabilities of Herbal Simples

**Published:** 1890-10-04

**Authors:** W. T. Fernie


### The History and Capabilities of Herbal Simples.

By W. T. Fernie, M.D.

*(Continued from page 391, Vol. VIII.)*

### XX.—CLUB MOSS.

When "The Mortal Struggle" was produced, amid weird scenic effects, at Portsmouth, by Mr. Vincent Crummles, with the aid of Miss Snevellicci, and the Infant Phenomenon, lurid lightning was much in request to astonish the natives. And it was sufficiently well simulated by igniting with a sudden flash and a hiss the highly-inflammable spores of Club Moss—lycopodium clavatum—projected against burning tow. The plant which affords these spores is of common growth in Great Britain, to be found on heaths and hilly pastures, particularly in the summer and autumn. It gets its first name, lycopodium, or wolf's claw, from the claw-like ends of its trailing stems; and is termed clavatum because of its inflorescence resembling a club. The liver-wort belongs to the same group of plants, and is so called from the liver shape of the thallus, and from its reputed efficacy in diseases of the liver. The spores of the club moss constitute a fine, pale, yellow, dusty powder, which is unctuous, tasteless, inodorous, and curiously medicinal. Each minute atom of this powder when seen under the microscope has a diameter of the eight hundredth part of an inch, and is in shape like a nut. If pounded in an agate mortar the nuts become fractured, and their contents, when a little water is added, are shown to be oil globules, wherein the curative virtues of the moss probably reside. Sugar of milk, when rubbed up for at least two hours with the broken spores, or a tincture made from them, with spirit of ether, will display their specific medicinal properties. This remedy is most remarkably useful for decay of physical strength, with mental and bodily prostration, sallow complexion, slow digestion, flatulence, heartburn, and water brash. It is said that whenever a fan-like movement of the wings of the nostrils can be observed, the whole group of these allied symptoms is curable by the club moss. Chemically, the oil globules of the spores contain alumina and phosphoric acid. Six drops of the ethereal tincture may be given for a dose, with a tablespoonful of cold water; or as much of the medicated sugar of milk as will lie on a sixpence, twice or three times in the day. Long ago the Cornish sages declared lycopodium to be good against all diseases of the eyes. When cutting it they first showed the knife to the moon, and then repeated these words:—

"As Christ healed the issue of blood,
Do thou cut what thou cuttest for good."

In the folk lore of Lancashire it is ordered that "at sundown, having carefully washed the hands, the club moss should be cut kneeling. It is next to be carefully wrapped in a white cloth, and subsequently boiled in water taken from the spring nearest its place of growth; and this may be afterwards used as an infallible fomentation. Or, the moss may be made into an excellent ointment with butter from the milk of a new cow. The common lycopodium bears also in some districts the name of Robin Hood's hatband. Its unmoistenable powder from the spores is a capital application to raw surfaces; and druggists put this in boxes containing pills to prevent them from adhering to each other. When subdivided again and again by repeated triturations it has proved actually beneficial for reducing the swelling and for diminishing the pulsation of aneurism occurring in the larger blood vessels of the heart. Of old it was said that the "club mosse, if hung in a vessell of wine that hath lost its vigour and virtue, so much as is convenient for the bignesse of the vessel, will in a short time recover them again; wherefore it is called the "Wine-herbe." Also the cup mosse will help the chin-cough in children effectually, if they drinke the powther thereof for certain daies together. To cure stone, half a dram of the mealy yellow powder, given during July, in any proper vehicle, is a noble remedy. And a decoction of the lycopodium made with water, or beer, will heal the plica polonica, a Polish disease, where the hair bulbs bleed, and the hair becomes plaited together. At the shops the powder of club moss—lycopodium clavatum—is sold as witch-meal, or vegetable sulphur. In the history of the Royal Society we are told that a certain dog went mad, and bit several other dogs belonging to the Duke of York; but by the timely use of a particular moss—the ground liverwort—they were all preserved from rabies. This moss grows commonly on our dry pastures and heaths. Its powder, mixed with black pepper, was recognized by the College of Physicians in 1721 as a medicine of singular value for preventing and curing hydrophobia. Dr. Mead, who had repeated experience of its virtue, declared that he never knew it to fail when used with the assistance of cold bathing. Again, another curative, British lichen, is found especially in Wales and Scotland, the Lichen Islandicus, better known as Iceland moss. It is an efficacious remedy for consumptive coughs and dysentery. Most probably the Icelanders were the first to learn its helpful properties. In two species of consumption this lichen best promises a cure: that with bleeding from the lungs, and that with profuse mattery phlegm. It is to be given as a decoction, made by boiling an ounce and a-half of the moss in a quart of milk, so that a tea-cupful may be drank frequently in the course of the day. In New England the generic term Moss is a cant name for money; perhaps as a contraction of "mopuses"; or as a play on the proverb, "A rolling stone gathers no moss."

